# Production systems and important antimicrobial resistant-pathogenic bacteria in poultry: a review

**DOI:** 10.1186/s40104-022-00786-0

**Published:** 2022-12-14

**Authors:** Philip H. W. Mak, Muhammad Attiq Rehman, Elijah G. Kiarie, Edward Topp, Moussa S. Diarra

**Affiliations:** 1 Guelph Research and Development Centre, Agriculture and Agri-Food Canada (AAFC), Guelph, ON Canada; 2 Department of Animal Biosciences, University of Guelph, Guelph, ON Canada; 3 London Research and Development Center, AAFC, London, ON Canada

**Keywords:** Antibiotic-free, Antimicrobial resistance, Conventional feeding, Organic, Poultry

## Abstract

Economic losses and market constraints caused by bacterial diseases such as colibacillosis due to avian pathogenic *Escherichia coli* and necrotic enteritis due to *Clostridium perfringens* remain major problems for poultry producers, despite substantial efforts in prevention and control. Antibiotics have been used not only for the treatment and prevention of such diseases, but also for growth promotion. Consequently, these practices have been linked to the selection and spread of antimicrobial resistant bacteria which constitute a significant global threat to humans, animals, and the environment. To break down the antimicrobial resistance (AMR), poultry producers are restricting the antimicrobial use (AMU) while adopting the antibiotic-free (ABF) and organic production practices to satisfy consumers’ demands. However, it is not well understood how ABF and organic poultry production practices influence AMR profiles in the poultry gut microbiome. Various Gram-negative (*Salmonella enterica* serovars, *Campylobacter jejuni/coli, E. coli*) and Gram-positive (*Enterococcus* spp.*, Staphylococcus* spp. and *C. perfringens*) bacteria harboring multiple AMR determinants have been reported in poultry including organically- and ABF-raised chickens. In this review, we discussed major poultry production systems (conventional, ABF and organic) and their impacts on AMR in some potential pathogenic Gram-negative and Gram-positive bacteria which could allow identifying issues and opportunities to develop efficient and safe production practices in controlling pathogens.

## Introduction

Poultry meat is an important animal protein and one of the most popular meat consumed by humans worldwide. Its consumption is projected to increase 17.8% by 2030 according to the OECD-FAO; the highest increase among all types of animal meats [1]. This significant increase is due to the rapidly growing poultry industry (annual global production of about 120 million tons) through genetic selection and the adoption of various measures to improve birds’ health and performance. Intensive poultry production driven by consumer’s demand continue to increase, especially in South America, Asia and Africa, possibly due to their recent change in diets for a more animal protein option [2]. Antibiotic use in the poultry industry revolutionized the therapeutic and economical gains by improving meat yield, bird’s health, and cost-efficient production. However, the growing concerns of the increasing prevalence of antimicrobial resistance (AMR), particularly against antibiotics of human importance have led to restrictions of antimicrobial use (AMU) in poultry in several countries. Despite these restrictions and the use of alternative production practices to reduce AMR in poultry, there have been multiple reports of AMR bacteria associated with poultry which present food safety concerns [3–5].

The gastrointestinal tract (GIT) plays a crucial role in poultry health as it provides the first-line of defense against foreign pathogens while also allowing nutrient absorption [6]. In addition to maintaining the homeostasis and nutrient processing, populations of different bacteria including *Lactobacillus*, *Clostridium*, *Ruminococcu*s, *Salmonella enterica* serovars, *Enterococcus* spp., and *E. coli* inhabit the GIT to constitute the gut microbiota. Through horizontal gene transfer of mobile genetic elements such as transposons and plasmids, the gut microbiota can be a reservoir for antimicrobial resistance genes (ARGs). The addition of antibiotics to poultry diets can modulate the gut microbiota by decreasing the pathogenic bacteria load, increased the intestinal nutrient absorption, and ultimately improved growth parameters [7]. Thus, it is important to understand how dietary practices modulate the poultry gut microbiome [6, 8].

Necrotic enteritis (NE) caused by a Gram-positive anaerobic spore-forming bacterium *C. perfringens* is one of the major poultry diseases costing $6 billion per year to the global poultry industry [9]. This bacterium, representing also a food safety issue, is widespread and commonly found in the environment and in the gut of humans and animals [10]. Sub-clinical NE lead to production losses associated with reduced weight gains and poor feed conversion ratios [11]. Intestinal damages induced by *C. perfringens* give bacteria access to the bile duct and blood stream, consequently damaging additional organs in birds [12]. Typical antibiotics such as avilamycin and bacitracin methylene disalicylate are used to prevent NE in poultry. Therefore, with the AMU restrictions in poultry, controlling this pathogen has become highly imperative not only for poultry gut health, but also from a food safety perspective [9]. Coccidiosis is also a major poultry intestinal disease caused by *Eimeria* spp. parasites which, invade and replicate in the intestine [13]. This parasitic disease causes annual losses greater than $600 million in the United States and $3.2 billion worldwide [13, 14]. *Eimeria* infections have also been associated with the promotion of NE [15, 16].

In poultry production, the type of feeding program is extremely important to ensure nutrient and health requirements are met. Feeding programs are selected based on cost efficiency, effectiveness to improve health and growth. Conventional feeding programs relied on AMU to improve growth performance while simultaneously preventing infectious diseases. However, increasing concerns over AMR resulted in the development and adoption of alternative productions known as antibiotic-free (ABF) productions, also called no antibiotic ever (NAE) or raised without antibiotic (RWA) and organic productions. Harmonization of the definitions used for the terms ABF, NAE and RWA is necessary, so “ABF” will be used for consistency in this review. Research on the effectiveness of alternative feeding programs to reduce AMR is needed to identify best production practices in preserving gut heath [17–19]. Substantial efforts are needed to not only understand the underlying mechanisms behind alternative feeding programs but also to understand their true impacts on AMR profiles in the gut microbiota.

In this review, we will discuss the significance of conventional, ABF and organic poultry productions by outlining the AMU concerns and the use of alternatives to antibiotics. Moreover, a summarization of some important antimicrobial agents and alternative products in poultry production is presented to highlight pathogenic bacteria of concern and opportunity for improvements for their efficient control while highlighting that AMR issues should be addressed by a “One Health” approach.

## Poultry feeding and production practices

Feed is a significant component in poultry production. Feed quality, nutrient composition, and consumption rate by birds are critical parameters to monitor for their health and productivity. Nutrients of poultry feed can be categorized into five different groups: carbohydrates, lipids, proteins, minerals and vitamins. High quantity and quality water is also essential. Each feed ingredient plays a vital role in either, energy acquisition and utilization, metabolism or health of poultry. For several years, sub-therapeutic levels of antibiotics have been used in broiler feed to maximize their productivity [20]. This practice contributed to meet the rapidly increasing chicken meat demands of the growing world population. However, AMR concerns led to alternative poultry feeding programs and production practices to be adopted. These alternative feeding programs include ABF and organic production which, in definition may vary by country around the world. However, organic and ABF poultry production requires alternative solutions to maintain or improve health. Moreover, the impact of alternative poultry feeding program on AMR deserve to be explored further.

### Conventional production

Conventional production practices were widely adopted to shape the livestock industry to what it is today. One key difference between conventional production and ABF or organic production is the use of antibiotics in healthy animal during conventional production. Justifications of antibiotics used in poultry production include growth promotion and prevention of important diseases. The World Health Organization (WHO) created a global critically important antibiotics (CIA) list that categorize antibiotics into three different classes based on their importance in human medicine; important, highly important, and critically important [21]. In addition to WHO, CIA lists were created by different countries with varying discrepancies in antibiotic classification. For example, the Public Health Agency of Canada CIA’s list classify antibiotics in four categories (I, II, III, and IV), where agents in category I are “very high importance” and those in category IV have “low importance” in human medicine. The Chicken Farmers of Canada (CFC) progressively eliminated the preventive use of Category I to III antibiotics by 2020. Accordingly, about 60% of broilers were raised without antibiotics in the United State of America in 2019 [22]. The trend to remove antibiotics from poultry production slowly increased in the past years, but concerns about bird's health and cost-efficiency remain to be issues in the development of antibiotic replacements.

### Organic production

Organic production typically raise animals naturally while maintaining optimal health, welfare and living conditions. More and more poultry producers are opting for organic production due to the sustainability and harmony with the environment. Each country has their own standards and regulations of organic production systems, such as the United States Department of Agriculture’s National Organic Program and the Standards Council of Canada’s CAN/CGSB-32-Organic Production Systems [23, 24]. Despite slight differences on the definition of organic production by country, the common rule of thumb requires free range systems (outdoor access on pasture), ecological sustenance, and compliance with all applicable regulatory requirements of substances [24]. However, the organic production requirements place heavy limitations that reintroduce health and management issues. For example, access to the outdoor pasture increases the risk of exposure to environmental microbes such as *Salmonella*, *Campylobacter* and *C. perfringens* [25], which are food safety and bird’s heath concerns. There is a perception among consumers that organically produced foods are more “natural” and therefore healthier than conventionally produced ones [26, 27]. However, organic broiler production costs were estimated to be 70%–86% higher than those of conventional production which consequently increased retail market costs of poultry products [28]. Furthermore, in organic production there is a high risk of colonization by pathogens that can cause diseases such as NE and coccidiosis in chicken (chicken health) and salmonellosis in human (food safety) [26]. Colonized pathogenic bacteria consequently may contaminate meat during processing. For example, it has been reported that organic broiler meat, at the end of processing after chilling, was more frequently contaminated with *Campylobacter* spp. than conventional broiler carcasses, possibly due to the organic bird’s free access to pasture where they could be more exposured to environmental of bacterial pathogens [29]. These authors also reported relative risks of 1.7 times increased risk of *Campylobacter* illnesses following consumption of contaminated organic broiler meat, compared to conventional broiler meat in Denmark. These reports on organic poultry production indicate that investigation are needed to develop cost-efficient methods to improve the gut health, reduce risks to consumers, and minimize negative impacts of production on the environment.

### Antibiotic-free production

Antibiotic-free production is similar to conventional production, with the exception of AMU as a prophylactic and for growth promotion. Thus, the potential health and production issues in ABF production requires alternative solutions (Section Alternatives to antibiotics in poultry production). Consumer perception and rising concerns about the food attributes direct attention to ABF-based poultry production; they are willing to pay premium prices for these products. However, the general consumer’s understanding of ABF is limited to positive advertisement and method of communication and do not discuss the negative issues of ABF production. According to Agri-Stats data in 2018, the mortality rate in ABF and conventionally raised birds were reported to be approximately 4.2% and 2.9%, respectively [30]. Growth promoting properties of antibiotics are used to evaluate the efficacy of alternatives products in controlling coccidiosis, NE and maintaining gut health [31]. In ABF production, vaccinations, high-quality feed and water, and heightened control of production environments are required to decrease stresses in birds while maintaining their health and performance [32, 33]. Despite these efforts, health and growth performance issues could arise due to ineffective prevention of diseases and the potential of negative side effects from antibiotic alternatives [17, 19, 32]. Moreover, even if these problems could be surmounted, it would be at the cost of expense [17]. For the prevention of coccidiosis, ABF production rely on vaccinations or chemically synthesized non-antibiotic coccidiostats [19]. However, studies indicated that, when compared to ionophores, chemical coccidiostats could promote the development of anticoccidials drug resistance and are typically more expensive than ionophores [19]. Surprisingly, it has also been observed that some chemical coccidiostats without the co-administration with antibiotics could induce NE [19].

### Alternatives to antibiotics in poultry production

Many different alternatives to antibiotics have been investigated in poultry production at an attempt to replicate their multifunctions. A list of alternatives investigated and/or used in poultry production include probiotics, prebiotics, organic acids, phytochemicals, vaccines, in-feed enzymes, and essential oils (Table 1). A description of each antibiotic alternative, their benefits in poultry health, and their effective function are briefly described below.

**Table 1:** Different categories of alternatives to antibiotics in poultry production

Product	Dosage range	Function	Reference
Probiotics	10^4^–10^9^ CFU bacteria	• Improve homeostasis of bacteria in intestinal microbiota • Inhibit pathogenic bacteria colonization • Improve growth performance of broilers • Improve/Strengthen immunity	[34–37]
Prebiotics	1–10 g/kg feed	• Inhibit pathogenic bacteria colonization • Improve digestibility • Catalyze the growth of healthy bacteria	[35, 38, 39]
Organic acids	0.5–3 kg/t feed; acidify water @ 5%	• Improve beneficial bacteria populations • Reduce pH to aid in digestion and reduce pathogenic bacteria	[18, 40–44]
Phytochemicals	0.3–60 g/kg feed	• Antioxidant • Antimicrobial • Antifungal • Anti-inflammatory • Anti-parasitic	[8, 45–47]
Vaccines	Varies by vaccine type	• Significantly improves immunity • Target-specific immunity	[19, 48]
In-feed enzymes	300–500 g/t feed 0.5–1 g/L water	• Improve digestibility, performance • Improve feed intake and body weight gain	[49–52]
Essential oils	0.1–0.5 g/kg feed	• Improve digestion • Improve blood circulation • Exhibit antioxidant properties • Reduce prevalence of pathogenic bacteria	[53, 54]

#### Probiotics

They are live microbial feed additives to help maintain intestinal microbial balance and benefit the host’s health [34–37]. Mostly identified as Gram-positive and some Gram-negative, Khan and Naz [37] reviewed commonly used probiotics including various *Lactobacillus* spp. (*L. bulgaricus, L. plantarum, L. acidophilus, L. helveticus, L. lactis, L. salivarius, L. casei, L. reuteri*), *Enterococcus faecium* and *E. faecalis*, *Streptococcus thermophilus*, and *Bacillus subtilis* in poultry. They function by interfering with the colonization of the gut by pathogenic bacteria through competitive exclusion [35–37]. Moreover, probiotics stimulate the immune system as shown by various studies correlating probiotic administration with elevated humoral and cellular immune responses by increasing T cell, CD^+^, B cells, and anti-inflammatory cytokine production [37, 55, 56]. Probiotics have been reported to improve body weight and feed-conversion ratio in commercial broilers [55]. However, the effectiveness of probiotics seems strain-dependent [36]. Probiotics naturally produce volatile fatty and organic acids and assist in digestion by breaking down insoluble fibers and improve nutrient absorption metabolism as well as lowering the pH of the gut to levels affecting pathogenic bacteria such as *E. coli* and *Salmonella* spp. [37]. Moreover, dietary probiotic *B. subtilis* was found to improve hen’s performance and egg quality at a lower dose while improving the protein quality in the eggs at a high dose [57]. In contrast, Sohail et al. [58] did not observe a positive impact on beneficial gut bacteria when investigating the effects of probiotics on the cecal and tracheal microbiota.

#### Prebiotics

They are carbohydrate-based polymers such as fructooligosaccharides (FOS), galactooligosaccharides, and mannanoligosaccharides (MOS) that function to promote beneficial bacteria in the gut, aid in digestion, and inhibit colonization by pathogenic bacteria [38]. Prebiotics are not utilized by the host but, they could be substrates for gut bacteria such as *Bifidobacterium* and other lactic acid bacteria. The prebiotic FOS indirectly alters the gut microbiota community through increased production of short-chain fatty acids (SCFA), some of which favour fermentation. The SCFAs are important in the GIT and immune function but, they also elicit acid stress to pathogenic bacteria [35, 38]. However, MOS can directly affect adhesion of pathogens such as *E. coli* and *Salmonella* to intestinal cells by binding to their flagella*,* which are important in their motility and attachment to intestinal epithelial cells [38]. Poultry cannot digest prebiotics because they are resistant to digestive enzymatic actions [38]. A study conducted by Ricke [39], indicated the potential of FOS as a beneficial prebiotic with its fermentation being limited to certain lactic acid bacteria. Prebiotics have been also reported to dose-dependently improve mineral absorption and immune function in poultry with minor adverse side effects [35].

#### Organic acids

These compounds have acquired a favourable reputation in the poultry industry due to their strong nutritional and antimicrobial properties. Moreover, organic acids have already been applied for feed preservation and performance enhancement in livestock production. These compounds have a carboxylic acid (R-COOH) group in common, in the simple monocarboxylic (formate, acetate, butyrate and propionate), hydroxyl (tartrate, citrate, lactate, and malate), or short-chain (fumarate, sorbate) form [19]. They acidify through lowering the pH of the gut to inhibit pathogenic bacterial growth, thus decreasing their prevalence and product contaminations while improving nutrient digestibility [40–42]. Butyrate and propionate have positive effects on the gut microbiota, such as down-regulation of the *Salmonella* pathogenicity islands which are important virulence-encoding genes in *Salmonella*. However, lactate has been identified to fuel *Salmonella* growth due to the utilization of the lactate-degrading respiratory lactate dehydrogenases LldD (converts *L*-lactate into pyruvate) and Dld (oxidates *D*-lactate to quinones) [59]. Moreover, acetate restored *barA* gene function in mutant *S. typhimurium barA*^*−*^; this gene encodes for the BarA sensor kinase important in the interaction with SirA to shift *Salmonella* from mobility to virulence [60].

#### Phytogenics

The immune-stimulatory potential of fruit products in poultry have been recently reviewed [61]. Berry fruit pomace, a major solid by-product from the juice industry, contains phenolic compounds such as flavonoids [45]. These compounds have antioxidant properties, which have been positively correlated with their antimicrobial activity [8, 46]. Islam et al. [8] investigated effects of low-bush blueberry pomace (LBBP) on gut microbiota of free-range birds when administered through feed and reported that *Lactobacillus* population in LBBP-fed birds were more abundant than those fed a control diet from 21 to 42 days old birds. Moreover, Das et al. [46] reported significant improvements of body weight along with improved intestinal health when supplementing feed with cranberry and blueberry by-products. These authors also reported that dietary cranberry products modulated the innate immune genes (caspase 1, apoptosis–related cysteine peptidase, chemokine receptor-5, interferon gamma, myeloid differentiation primary response gene 88, and Toll-like receptor 3) and suppressed proinflammatory cytokines in broilers [46].

#### Essential oils

They are known to have antioxidant and antimicrobial properties [53]. With the increasing demand for ABF production, essential oils as feed additives in poultry production have become more popular. Dietary cinnamaldehyde (Cinnamomum) and citral (3,7-dimethyl-2-6-octadienal) were found to reduce the severity and incidence of NE and coccidiosis [54], and the proportion of antibiotic resistant *E. coli* while improving the intestinal digestibility, the overall performance and the meat quality in broilers [54, 62]. Broilers fed a natural blend of essential oils (basil, caraway, lemon, laurel, sage, thyme, oregano, tea) showed a significant increase in weight gain and feed-to-gain ratio, with an overall increase in carcass weight, breast weight, and relative percentage of breast meat [63].

#### Enzymes

These biological catalysts are typically administered to assist in digestion of certain feedstuffs [49]. Enzymes for poultry feed are mostly derived from fungi and bacteria, with xylanase and glucanase constituting majority (> 80%) of the global market for carbohydrase [49]. In-feed enzymes are more recently investigated in broiler production due to the rising costs of feed ingredients such as soybean meal and corn, so cheaper feed alternatives that contain non-starch polysaccharides (NSP) were considered. Since NSP are not completely digestible in broilers, in-feed enzymes are added in response to the adverse effects of NSP [64]. The impacts of enzymes on ruminant performance and health have been studied extensively but such extensive studies are lacking in non-ruminants including poultry. However, it is well understood that feed enzymes are required to fully degrade certain chemical bonds of feedstuffs allowing access to amino acids and minerals where the host normally could not access alone [50]. One naturally available enzyme in poultry is phytase, which hydrolyzes phytic acids allowing the host to have a better access to phosphorus; which is one of the most expensive nutrients of feed [51]. Contrasting studies conflict on the effect of enzymes on growth rate and feed intake, but other studies all agreed on an increased nutrient digestibility when administering in-feed enzymes [65, 66].

Several health and economic challenges arise when implementing ABF productions using the alternative products. Despite promised beneficial activities, the efficacy of antibiotic alternatives is quite variable by study. It is hypothesized that combination of these individual alternatives may provide optimal activities. This probably could explain in the decrease of overall performance (average daily gain, feed conversion ratio, meat yield, mortality, etc.) with a single alternative product when compared to traditional antibiotics [17, 19]. Requiring more feed, time, and space to mitigate these deficits will be more costly compared to antibiotics, as well as increased carbon foot-printing. Nevertheless, assuming that all of these factors were somehow managed, readjustment of production practices would result in higher costs than conventional production which will affect the price of broiler meat in retail markets. Furthermore, the impacts including AMR, of these alternatives to antibiotics in production deserve investigations under a “One Health” perspective.

## Antimicrobial use and concerns

Antimicrobials significantly contribute to the treatment and prevention of infectious diseases, the improvement of poultry performance, and overall yield [67–69]. However, concerns arose regarding the excessive AMU with the most significant issues being the emergence and spread of AMR among bacteria [70, 71] through selection of antibiotic resistant strains and dissemination of genes conferring AMR by horizontal gene transfer [72–74]. Antimicrobial resistance causes loss in therapeutic efficacy of antibiotics resulting in increased morbidity and mortality rates due to infectious diseases in both animals and humans [70, 75, 76] thus constituting significant socio-economic and public health issues.

Several studies reported links between AMU in food animal production and the prevalence of AMR in bacteria [77, 78]. The prevalence of AmpC-like β-lactamase *bla*_CMY-2_ genes harboring *Salmonella enterica* and *E. coli* from infected humans were positively correlated to ceftiofur-resistant *Salmonella* and *E. coli* from chicken meat [79]. Action plans to decrease AMR are being implemented under a “One Health” approach in several countries. Moreover, WHO responded with a Global Action Plan, outlining important objectives to succeed in the fight against AMR. However, it was reported that 73%–80% of all antibiotics sold worldwide were used specifically for food animal production [2, 80]. Due to the AMR crisis, several poultry producing countries including Canada, United States of America, Brazil, China and the European Union have restricted the use of antibiotics as growth promoters and for disease prevention in animal production [81, 82].

## Some antimicrobials used in conventional poultry

Typical conventional broilers are raised in barns from hatching to 36–42 d during which each bird can consume 3.2 to 4.0 kg of feed to reach a body weight of about 1.8 to 2.2 kg [7]. Conventional broiler feeds which, are generally formulated according to the growth phases (starter, grower and finisher), are mainly grain-based to which protein, minerals and vitamins are added for nutritional requirements along with antibiotics. Major antibiotics used for therapy in poultry feed include aminoglycosides (gentamicin, neomycin, spectinomycin, and streptomycin), β-lactams (penicillin and amoxicillin), sulfonamides and tetracyclines [83]. Antibiotics that have been used for disease prevention and growth promotion in poultry include glycolipids (bambermycin), polypeptides (bacitracin), ionophores (salinomycin), streptogramin (virginiamycin), and orthosomycin (avilamycin). However, the accurate estimates for the number and amount of antibiotics used in poultry production systems globally are lacking [2, 84]. Some common antimicrobials used for disease prevention and growth promotion in broiler production are discussed below.

### Avilamycin

Is an orthosomycin antibiotic from *Streptomyces viridochromogenes*, targeting Gram-positive bacteria such as *C. perfringens* to prevent NE in broiler chickens [85]. It has been used as a growth promoter in poultry production [67, 68]. Avilamycin is unclassified in the WHO list of critically important antimicrobials for human medicine (WHO CIA), justifying its use in current poultry production [21]. Avilamycin inhibits bacterial protein synthesis by binding to their 50S ribosomal subunit’s helices 89 and 91 interfering thus, with tRNA and initiation factor 2 [85, 86]. Resistance to avilamycin can be mediated by mutations in helix 89 and 91 of the 23S rRNA [86] or in the ribosomal protein L16 [87, 88]. Various avilamycin resistance bacteria such as *Enterococcus faecium* have been reported in broiler fecal from several farms in Denmark and France [89, 90].

### Bambermycin

Is also known as flavomycin, flavophospholipol or moenomycin. This phosphoglycolipid antibiotic originating from various strains of *Streptomyces* including *S. bambergiensis* and *S. ghanaensis* is not categorized in the WHO/CIA list as important in human medicine, but used in poultry production [91]. Targeting primarily Gram-positive, bambermycin inhibits peptidoglycan synthesis through disruption of the penicillin-binding proteins (PBPs) transglycosylase activities, affecting bacterial cell wall production [67, 92]. Resistance mechanisms against bambermycin are not fully understood. However, due to its similar mechanism of actions to β-lactam (targeting PBPs), mechanism of actions for resistance to bambermycin could be related to β-lactam resistance.

### Bacitracin

This cyclic polypeptide antibiotic produced by *Bacillus licheniformis* and *B. subtilis* strains is categorized as important in the WHO/CIA list [93]. Primarily targeting Gram-positive bacteria, bacitracin interferes with the dephosphorylation function of C_55_-isoprenyl pyrophosphate which is a lipid carrier involved in bacterial peptidoglycan synthesis [93, 94]. Dephosphorylation of C_55_-isoprenyl pyrophosphate results in the prevention of transport of *N*-acetylglucosamine (NAG) and β-(1–4)-*N*-acetylmuramic acid (NAM) to build the peptidoglycan wall [95]. Bacitracin is widely used for prevention of NE in broiler production. It is typically administered in the form of bacitracin methylene disalicylate (BMD) or zinc-bacitracin (BACN-Z) [95, 96]. Bacitracin resistance has been correlated with the presence of the *bcrR* gene encoding a unique membrane-bound one-component system through a putative ATP-binding cassette (*bcrAB*) transporter [97, 98].

### Monocarboxylic polyether ionophores

Salinomycin, narasin, and monensin belonged to this group and are unclassified in the WHO/CIA list [21]. They are produced by *Streptomyces albus*, *S. aureofaciens*, and *S. cinnamonensis,* respectively. Salinomycin use dates back to its discovery in 1974, demonstrating effectiveness against Gram-positive bacteria and coccidiosis [99]. Salinomycin has been reported to improve bird's performance and prevent infectious diseases presumably by altering the composition and activities of intestinal microflora in broiler. Historically, salinomycin was considered less important in human medicine, however, it is now a well-known inhibitor of human cancer stem cells and has been suggested to suppress the growth of colorectal cancer by disrupting the β-catenin/TCF complex [100]. Ionophores facilitate the transport of cations into target organisms such as *Eimeria* spp. by disrupting their osmoregulation [101]. The mechanisms of resistance against ionophores are not fully understood, but ionophores are suspected to be excluded from cell membranes by an extracellular polysaccharide called glycocalyx [102].

### Mechanisms in poultry

Antibiotics and their alternatives on livestock or poultry may have complex direct or indirect mechanism of actions [103]. Antibiotics in broiler diets can alter the composition and activities of the bird’s gut microflora by killing, inhibiting or promoting bacteria resulting in improved health and advantageous economic outcomes [67]. More research is needed to systematically evaluate effects of specific antibiotics on the overall dynamics of gut microflora as well as on the distribution of ARGs among bacteria in chicken. Justifications for certain antibiotics used for growth promotion (i.e. avilamycin and bambermycin) need to be established as concerns for cross-resistance and co-selection to traditional antibiotics important in human medicine has been legitimated. An example includes bacteria showing resistance to avilamycin and cross-resistance to evernimicin, an antibiotic which inhibits the 50S ribosomal subunit formation in *Staphylococcus aureus* cells and are used to treat humans [86, 90, 104, 105]. Isolates of *S. enterica* serovar Heidelberg showed resistance to the third-generation cephalosporin ceftiofur (used in animals only) and ceftriaxone (very important in treating bacterial infections in human) [79]. This raised significant concerns to human health due to possible cross-resistance between third-generation cephalosporins such as ceftiofur, ceftriaxone and cephamycin [106, 107].

**Fig. 1:**
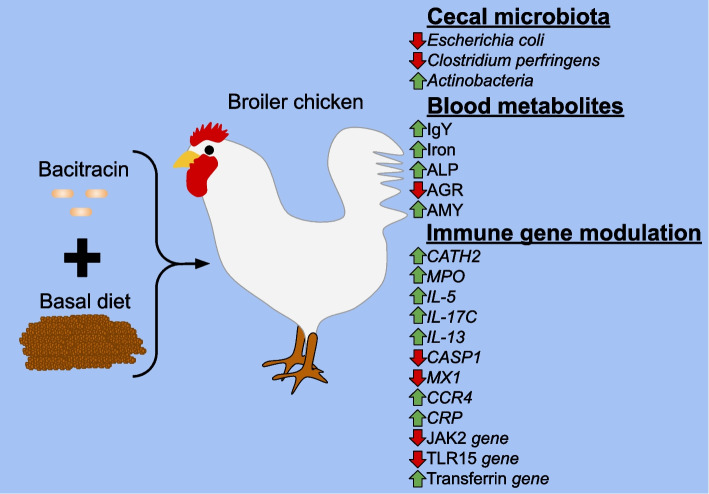
Bacitracin effect on broiler chicken cecal microbiota, blood metabolites, and immune gene modulation. ALP: Alkaline phosphatase; AGR: Albumin-globulin ratio; AMY: Amylase; CATH2: Cathelicidin antimicrobial peptide; MPO: Myeloperoxidase; CASP1: Caspase 1; MX1: Myxovirus resistance 1; CCR4: Chemokine receptor 4; CRP: C-reactive protein; JAK2: Janus kinase 2 "Updated from Das et al. [47]"

Antibiotics may also have important effects on animal physiology that are not studied in detail despite their significant effects against bacteria. Thus, it is important to study their effects on host’s physiology and immunology to better understand their interactions and design better alternative production practices. An example of this interaction was reported with bacitracin, showing modulation of the poultry blood serum metabolite profiles through increasing the alanine aminotransferase and decreasing albumin/globulin ratio levels (Fig. 1) [46]. The reduced albumin/globulin ratio in bacitracin-fed birds could indicate acute or chronic inflammatory processes due to an elevated globulin level or other uncharacterised mechanisms. Interestingly, Das et al. [47] reported a 22.55-fold, 12.34-fold, and 7.97-fold expression in *CATH2*, *MPO,* and *IL-5* genes respectively, in immune organs of bacitracin-fed birds compared to a control diet (Fig. 1). Understanding the mechanisms of how other health management practices such as how prebiotics, probiotics and vaccines interact with broilers is critical in the development of improved production systems [90, 108].

## Poultry production systems and AMR bacteria

**Fig. 2:**
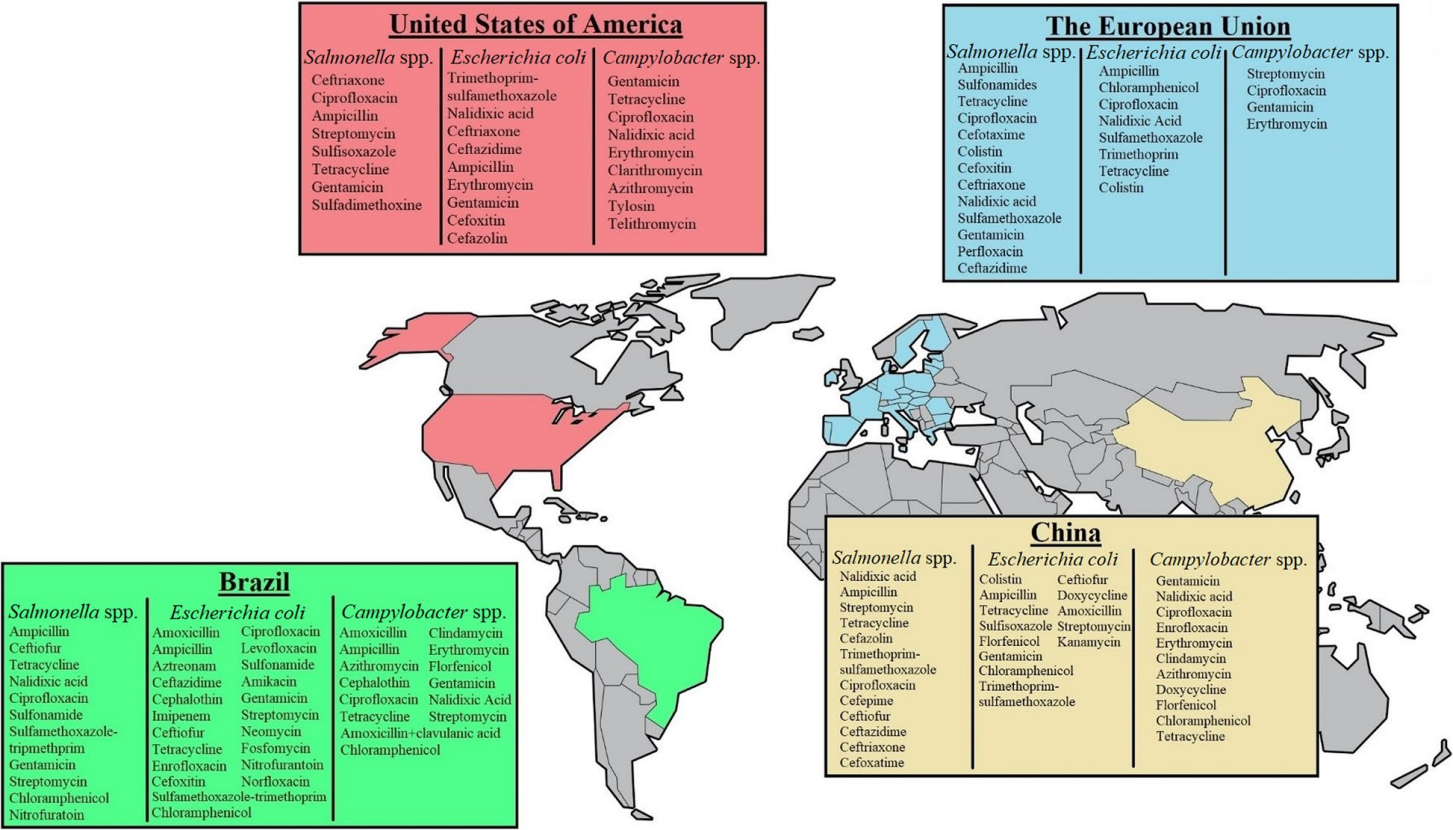
Antibiotic resistance in *Salmonella*, *E. coli* and *Camplylobacter* spp. reported in major poultry production countries from 2000 to 2020. Information obtained from: Brazil [41, 121–129], Europe [130–132], USA [133–139], China [140–143]

**Fig. 3:**
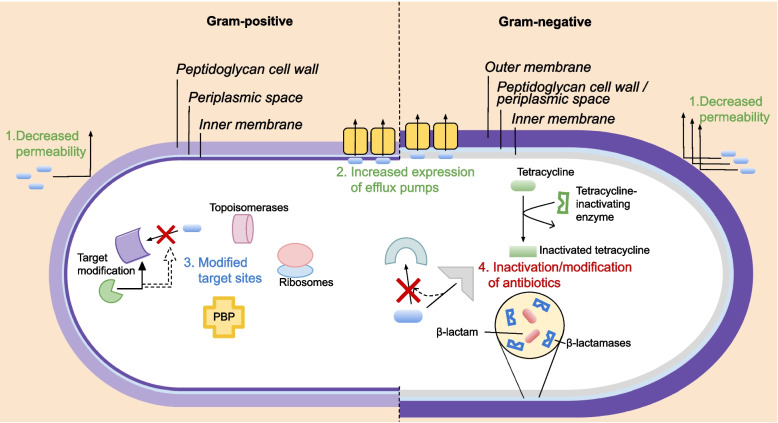
Schematic representation of Gram-positive and Gram-negative antimicrobial resistance mechanisms. 1. Decreased permeability of antibiotics due to outer membrane, 2. Increased expression of efflux pumps, 3. Modification/mutation of target sites (i.e. topoisomerases, ribosomes, penicillin-binding proteins (PBPs), fluoroquinolones, etc.), 4. Inactivation and modification of antibiotics via inactivating enzymes (i.e. β-lactamases, tetracycline-inactivating enzymes)

Various studies have been conducted on the AMU in food animals and their consequences on AMR in foodborne bacteria [4, 7, 90, 97, 109–111]. Here, the impacts of conventional and alternative production systems on AMR are presented to clarify concerns moving from conventional to ABF or organic production and the consequential effect on AMR in the birds and their products. The most common AMR reported in poultry pathogenic bacteria such as *S. enterica* serovars*, Campylobacter jejuni, E. coli, Staphylococcus aureus,* and *C. perfringens* are discussed (Table 2). Antibiotic-resistant non-typhoidal *S. enterica* serovars, *E. coli,* or *Campylobacter* spp. can infect humans through contact or consumption of contaminated food (food safety). A positive association between consumption of antibiotics in poultry and corresponding antibiotic resistance in some bacteria has been reported in Europe [83]. It is clear on how conventional production affects AMR; however, it is unclear on how organic or ABF production affects AMR. There are limited studies on AMR in organic and ABF systems. A summary of antibiotic-resistant *E. coli, Salmonella* spp., and *Campylobacter* spp. reported worldwide in poultry is presented in Fig. 2 and a schematic overview of mechanisms of AMR in bacteria is presented in Fig. 3. Organic and ABF production practices have been adopted to reduce AMU; however, various multi-drug resistant (MDR) bacteria from poultry raised from these alternative production practices have been reported (Table 3). Understanding on how these production practices truly affect the AMR profiles of the poultry gut microbiome (resistome) need to be established.

**Table 2:** Important antimicrobial-resistant bacteria reported in conventional poultry

Bacterial species	Disease	Antimicrobial resistances	Reference
*Salmonella* spp.	Salmonellosis, gastroenteritis, bacteremia, enteric fever fowl typhoid, pullorum disease	Streptomycin, tetracycline, sulfamide	[112]
		Ampicillin, amoxicillin-clavulanic acid, ceftiofur, cefoxitin, ceftriaxone	[113]
		Amoxicillin, ceftiofur	[110]
*Campylobacter jejuni*	*Campylobacter*iosis, gastroenteritis, bacterial diarrheal, Guillain-Barré syndrome	Ampicillin, nalidixic acid, tetracycline	[114]
		Quinolone, tetracycline, amoxicillin	[115]
*Escherichia coli*	Colibacillosis, bacteremia, UTI, meningitis, pneumonia, cholecystitis, diarrhea, cholangitis, septicemia, pericarditis, airsacculitis, salpingitis, peritonitis, cellulitis	Tetracycline, streptomycin, sulfonamides (sulfisoxazole), trimethoprim, ampicillin	[112]
		Tetracycline, nalidixic acid, ciprofloxacin, sulfonamides, chloramphenicol, quinolones and fluoroquinolones, β-lactams, ampicillin	[116]
		Ampicillin, cephalothin, ciprofloxacin, doxycycline, streptomycin	[27]
		Tetracycline, amoxicillin, ceftiofur, spectinomycin, sulfonamides	[7]
		Amoxicillin, ceftiofur, tetracycline	[110]
		Ampicillin, cephazolin, streptomycin, tetracylines	[117]
*Staphylococcus aureus*	Pulmonary infections, heart/bone/joint infections, gastroenteritis, osteomyelitis, septic arthritis, abscesses, furuncles, cellulitis, meningitis, UTI arthritis, tenosynovitis, osteomyelitis, omphalitis	Clindamycin, doxycycline, oxacillin	[27]
		Methicillin, amoxicillin, ampicillin, oxacillin, penicillin, ceftiofur, oxytetracycline, tetracycline	[118]
*Clostridium perfringens*	Necrotic enteritis, clostridial myonecrosis/gas gangrene	Tetracycline, bacitracin	[119, 120]
*Enterococcus faecium*	Endocarditis, UTI, prostatis, intra-abdominal infection, cellulitis, wound infection, bacteremia	Lincomycin, bambermycin, bacitracin, tetracycline, ciprofloxacin, erythromycin, kanamycin, penicillin, tylosin, streptomycin, vancomycin, gentamycin, streptogramins, avilamycin	[4, 88]
*Enterococcus faecalis*	Endocarditis, UTI, prostatis, intra-abdominal infection, cellulitis, wound infection, bacteremia septicaemia, endocarditis, salpingitis, arthropathy, amyloidosis	Lincomycin, quinupristin/dalfopristin, tetracycline, bacitracin, erythromycin, tylosin	[4]

**Table 3:** Important antimicrobial resistant bacteria reported in organic poultry

Bacterial species	Antimicrobial resistances	Reference
*Salmonella* spp.	Streptomycin, tetracycline, kanamycin	[144]
	Amoxicillin/clavulanic acid, ampicillin, azithromycin, cefoxitin, ceftiofur, ceftriaxone, chloramphenicol, nalidixic acid, streptomycin, sulfisoxazole, tetracycline, trimethoprim/sulfamethoxazole	[145]
*Campylobacter* spp.	Tetracycline	[146]
*Escherichia coli*	Ampicillin, cephazolin, streptomycin, tetracycline	[117]
	Ampicillin, erythromycin	[134]
	β-lactams	[147]
*Staphylococcus aureus*	Clindamycin, oxacillin	[27]
*Clostridium perfringens*	Not available	
*Enterococcus* spp.	Streptomycin, erythromycin	[148]

### AMR in Gram-negative bacteria

A wide variety of Gram-negative bacteria can cause diseases in poultry (*E. coli*, *Salmonella Pullorum/Gallinarum*, *Gallibacterium anatis, Pasteurella multocida, Klebsiella* spp.) and foodborne illness in human (*S. enterica* serovars and *Campylobacter jejuni/coli)*. The complexity of the Gram-negative bacterial cell surface provides intrinsic resistance against various antibiotics [149]. Antibiotics such as cephalosporins, carbapenems and fluoroquinolones are effective against Gram-negative bacteria, however, Gram-negative bacteria resistant to these antibiotics have been reported (Fig. 2). Resistance in Gram-negative bacteria can be acquired and/or intrinsic, with an overview of mechanisms of resistance being presented in Fig. 3.

#### *Escherichia coli*

*Escherichia coli* is a commensal bacterium of the gastrointestinal microflora. Some strains of this bacterium are known to cause diseases such as colibacillosis, cystitis, pyelonephritis, sepsis/meningitis, and gastroenteritis in both humans and animals due to the presence of various virulence factors [150]. The extraintestinal pathogenic *E. coli* (ExPEC) strains are epidemiologically and phylogenetically distinct from both intestinal pathogenic and commensal strains [151]. Avian pathogenic *E. coli* (APEC) is an ExPEC responsible for significant economic losses in the poultry industry [152] and was suggested to cause urinary tract infections and meningitis in humans, highlighting their safety risks [153].

In conventional poultry production, AMU has been correlated to the increased prevalence of AMR in *E. coli* [7, 62, 150, 154]. Antibiotics belonging to cephalosporins, quinolones, aminoglycosides and sulfonamides are used against *E. coli* infections. Multiple resistance to amoxicillin–clavulanic acid, ceftiofur, ceftriaxone, cefoxitin, gentamicin, sulfonamide and tetracycline in commensal and APEC isolates have been documented in poultry [7, 62, 150, 154]. Antibiotic resistance genes *bla*_TEM_, *bla*_SHV_, *bla*_CMY-2_, *aac (3)-Via*, *aadA1*, *aph (3)-Ib*, *aph (6)-Id*, *sul1, sul2, tet(A*) and *tet(B),* were observed in corresponding resistance phenotypes. Mobile genetic elements including pAPEC-O2-R, *IncA/C2* and *IncI1* plasmids as well as Class I integrons carrying antibiotic resistance genes *tet(A), sul1*, and *bla*_TEM_ able to be transferred to a recipient bacterium have been also observed in *E. coli* from broilers [150]. Dietary bambermycin, penicillin, salinomycin, and bacitracin or a combination of salinomycin plus bacitracin in broiler resulted in a higher incidence of ceftiofur, spectinomycin, and gentamicin resistance in *E. coli* isolates than those from the non-medicated feeds [7]. Interestingly, these authors observed a higher prevalence of *sul1*, *aadA*, and Class 1 integrons in salinomycin-fed chickens than in control or other treatment groups [7]. Regardless of antimicrobial feeding, they also noted multiple antibiotic-resistant *E. coli* isolates harboring corresponding genes such as *bla*_CMY-2_, *bla*_TEM_, *tetB*, *sul1*, and *aadA* [7]. Oral administration of tetracycline was not found to significantly induce changes in the chicken cecal bacterial community, however, population of tetracycline resistance *E. coli* harboring *tet(A)* or *tet(B)* increased [155]. Extended-spectrum β-lactamase-producing *E. coli* and *Klebsiella pneumoniae* were reported in local and imported chicken meat [156]. From conventional commercial broiler chickens, *E. fergusonii* harboring resistance to ampicillin, streptomycin and tetracycline were isolated, but the antibiotic usage from the studied farm was unknown [157]. These authors reported that 94.5% of the ampicillin-resistant *E. fergusonii* isolates tested contained the β-lactam *(bla*_CMY-2_), aminoglycoside (*aadA1*, *strA*, *strB*), trimethoprim (*dfrV*, *dfrA1*), tetracycline (*tet*(*A*), *tet*(*B*), *tet*(*C*), *tet*(*E*)), and sulfonamide (*sul1*, *sul2*) resistance genes [157].

As organic and ABF poultry production systems are becoming popular, their efficacy to reduce AMR deserves investigations. Recently, the prevalence of antimicrobial resistant commensal *E. coli* was found to be lower in organic and ABF broilers compared to conventionally produced ones [117]. However, *E. coli* isolated from organic, ABF and conventional production systems demonstrated high frequencies of resistance (> 50%) to ampicillin, cefazolin, sulfonamides, streptomycin and tetracycline [117]. It has been reported that *E. coli* isolates from conventional poultry meat were more MDR than those from organic poultry meat [27]. Even though the possible effectiveness of organic acids as an alternative to antibiotics have potential, some bacteria such as *E. coli* and *Lactobacilli* can survive in acidic environments due to their innate acid resistance properties [158]. Davis et al. [133] reported no significant difference between conventional, RWA and organic productions for the overall prevalence of antimicrobial resistant *E. coli* in chicken and turkeys, although differences were noted in specific antibiotic resistant phenotypes. Moreover, a higher prevalence of resistant *E. coli* to ampicillin, ampicillin-sulbactam, cefazolin, cefoxitin, ceftriaxone, and trimethoprim-sulfamethoxazole was found in conventionally raised turkeys compared to organic and RWA produced ones [133]. However, Sanchez et al. [134] reported a 56.2% and 60.7% resistance to ampicillin in *E. coli* when fed conventional and ABF, respectively [134]. In the Netherlands, extended-spectrum β-lactamase (ESBL)-producing *E. coli* harboring *bla*_CTX-M-1_ and *bla*_CMY-2_ genes have been isolated from commercial organic broilers [147].

#### Non-typhoidal *Salmonella enterica* (NTS)

In food production animals, NTS induces diarrhea with fever, anorexia, and dehydration. However, poultry can be asymptomatic carriers of these pathogens and exposure to antimicrobials could promote AMR isolates that may be transmitted to humans [5, 159]. It has been estimated that NTS causes 1.35 million infections (212,500 infections due to AMR isolates), 26,500 hospitalizations, and 420 deaths each year in the United States, resulting in about $400 million in direct medical costs [160]. Most cases of human salmonellosis (diarrhea) do not require antibiotic treatments. However, severe cases in elderly, children, or those with underlying comorbidities may require antimicrobial treatment, such as invasive infection resulting in life-threatening bloodstream infections. Recommended antimicrobials to treat NTS include ciprofloxacin, ceftriaxone, trimethoprim/sulfamethoxazole, or in last resort cases, amoxicillin and carbapenem. Several NTS serovars from conventional poultry farms showing resistance to ampicillin, amoxicillin-clavulanic acid, ceftiofur, cefoxitin, and ceftriaxone were reported [113]. Moreover, genes associated with aminoglycoside (*aadA1*, *aadA2*, *strA*), β-lactams (*bla*_CMY-2_, *bla*_SHV_, *bla*_TEM_), tetracycline (*tet(A)*, *tet(B)*) and sulfonamides (*sul1*) were detected in these *Salmonella* isolates [113]. Fosfomycin is an antibiotic approved to treat urinary tract infection cases, but its use is restricted in poultry production. However, a fosfomycin resistance gene, *fosA7*, was identified in *S. enterica* serovar Heidelberg isolated from conventional broilers [161]. In China, it has been reported that 60.1% of all non-duplicate *Salmonella* isolated from retail raw poultry meats were MDR to at least three different classes of antimicrobials, which included nalidixic acid, ampicillin and streptomycin [140]. Co-resistance to ciprofloxacin and ceftriaxone was most prevalent (84.1%) in *S. enterica* serovar Indiana [140]. A clonal group of *S. enteritidis* known as SE86, a frequently identified poultry *Salmonella* isolate in Brazil associated with foodborne outbreaks, has been reported to be resistant to ciprofloxacin (41.9%) and sulfafurazole (75%) [121]. A persistent septicemia causing *S. enteritidis* (SE_TAU19) resistant to nalidixic acid and sulfadimethaxine was reported [162]. Quesada et al. [130] found the *mcr-1* gene (colistin resistance) in *E. coli* and *S. enterica* from poultry and swine. From commercial poultry farms, Liljebjelke et al. [135] reported MDR *Salmonella* resistant to streptomycin, gentamycin, sulfadimethaxine, trimethoprim, and tetracycline. A recent report from Europe indicated no associations between consumption of cephalosporins and quinolone in poultry and resistance to these antibiotics in *Salmonella* isolates from humans [83].

A few studies on AMR *Salmonella* have been conducted in organic and ABF poultry production systems [144, 145, 163, 164]. The prevalence of amoxicillin-clavulanate, ampicillin, cefoxitin, ceftiofur, and ceftriaxone-resistant *Salmonella* isolates from large-scale organic poultry production farms was significantly lower than isolates from conventional broiler production [144, 163]. In contrast, a significantly higher AMR *Salmonella* isolates were found in ABF broiler than in those from conventional production [145]. *Salmonella* isolated from US conventional retail poultry meat showed 2.6 times higher resistance prevalence compared to those from organic retail meats [164].

#### *Campylobacter spp.*

*Campylobacter* spp. (*C. jejuni* and *C. coli*) are important foodborne pathogenic bacteria associated with poultry. These bacteria are microaerophilic and certain environmental stresses such as exposure to air, drying, low pH, and prolonged storage can be detrimental to their survival. In humans, these pathogens cause a self-limiting diarrheal disease from improperly prepared or contaminated food, including poultry products. In the United States, *Campylobacter* are responsible for an estimated 2 million cases of gastroenteritis annually. Antibiotics belonging to the macrolides (erythromycin), fluoroquinolone, tetracyclines and aminoglycosides (gentamicin) classes are used against Campylobacteriosis [165]. Despite an interprovincial observed difference in the AMR profile, a Canadian study suggested that AMR observed in *Campylobacter* isolates from chicken could be originated from upstream [166]. These authors reported more quinolone-resistant *Campylobacter* isolated in British Columbia, while those isolated in Quebec and Ontario provinces were predominantly resistant to tetracyclines, macrolides, ketolides, and lincosamides [166]. The emergence of fluoroquinolone resistance among *Campylobacter* from poultry led to the restriction or ban of sarafloxacin and enrofloxacin used in poultry [167]. An extremely high (88.6%–100%) prevalence of resistance to macrolides, tetracyclines, quinolones, and chloramphenicol was found in *Campylobacter* spp. isolated from conventionally-raised broiler chickens [168]. Moreover, it was reported that majority of the *Campylobacter* spp. isolated from turkeys were resistant to over seven antimicrobials [169]. Correlation between the prevalence of macrolide-resistant *Campylobacter* and the use of macrolides along with a trend of increasing prevalence of *erm(B)* gene in isolates were observed in poultry [170, 171].

Limited studies investigated the presence of antibiotic resistant *Campylobacter* in ABF and organic poultry productions. However, a study reported a significantly lower fluoroquinolone resistant *Campylobacter* prevalence (< 2%) in organic than in conventional (46%) poultry farms [136]. Susceptibility test of 157 *Campylobacter* isolates from organic (*n =* 77) and conventional (*n =* 80) chickens showed that all organic isolates were sensitive to all antibiotics, except two that were resistant to tetracycline, while resistance to quinolones and tetracycline were observed among the 80 isolates from conventional chickens [172]. Despite limitations, fecal, carcasses, equipment, water and air sample analyses from organic and conventional processing methods suggested that raising birds without the use of antimicrobials is not effective in decreasing the incidence of AMR *Campylobacter* in poultry products [146]. However, effects of Canadian AMU reduction on AMR in major poultry-associated foodborne pathogenic Gram-negative bacteria (*Salmonella*, *Escherichia coli*, and *Campylobacter*) showed the potential for progressive transitions from conventional to antibiotic-free broiler production [173]. The above review indicated the lack of studies investigating AMR in different production and alternative gut health management practices in poultry.

### AMR in Gram-positive bacteria

Gram-positive bacteria including *Enterococcus* spp., *Staphylococcus* spp.*,* and *C. perfringens* are common in poultry and can be commensal or pathogenic*.* According to a meta-analysis performed by Cardinal et al. [174], the most frequently used antibiotics in broiler during the last 30 years predominately targeted Gram-positive bacteria. As shown in Fig. 3, these bacteria lack an outer membrane which is compensated by a thicker (30–100 nm) peptidoglycan cell wall [175]. Examples of major AMR Gram-positive bacteria of concerns include methicillin-resistant *S. aureus* (MRSA), vancomycin-resistant *S. aureus* (VRSA), MDR *Streptococcus pneumoniae*, and vancomycin-resistant *E. faecium* (VRE). Several MDR Gram-positive bacteria have been isolated from conventional, organic and ABF poultry productions [4, 27, 97, 176, 177].

#### *Enterococcus spp.*

They were initially described as *Micrococcus* and fecal streptococci more than 113 years ago [178] belonging to the Firmicutes phylum of Bacilli class, Lactobacillales order, Enterococcaceae family, and *Enterococcus* genus (more than 40 species). *Enterococcus* spp. particularly *E. faecalis*, *E. faecium* and *E. cecorum* have been associated with diseases in both human and poultry [179, 180]. Therapeutic options of enterococcal infections include a combination of penicillin (ampicillin or penicillin) and aminoglycoside (gentamicin or streptomycin), vancomycin and quinupristin-dalfopristin (for *E. faecium* only). Newer antibiotics (linezolid, daptomycin, tigecycline and 5^th^-generation cephalosporins) or older antibiotics (chloramphenicol, doxycycline, minocycline and nitrofurantoin) have been also considered to fight against *Enterococcus*. However, these bacteria are characterised by intrinsic resistance to important antibiotic classes and to tolerate low concentrations of β-lactams, quinolones, aminoglycosides, and lincosamides, as well as being able to metabolise preformed folic acid (trimethoprim and sulphonamides). Furthermore, *Enterococci* have developed a high ability to acquire exogenous resistance genes via conjugative transposons and plasmids [181]. In conventional production, AMU has been associated with increased AMR *Enterococci* isolates and a potential zoonotic transmission of AMR isolates has been suggested [97, 182]. Association between the use of virginiamycin and virginiamycin-resistant *E. faecium* was reported in a surveillance study conducted by Aarestrup et al. [183]. Subsequent studies reported a strong correlation between the presence of streptogramin resistance genes in *E. faecium* in humans and the use of virginiamycin [81]. A case-control study in France determined a significant correlation (risk factor of 2.3) between the prevalence of avilamycin-resistant *E. faecium* and avilamycin use during broiler production [90]. Avilamycin-resistant *E. faecium* has also been reported to be cross-resistance to evernimicin [86, 90, 104, 105] and demonstrated MDR to other antibiotics such as penicillin, tetracycline, streptomycin and erythromycin [111]. All avilamycin-resistant *E. faecium* isolates contained the *emtA* gene encoding a methyltransferase which inhibits avilamycin and evernimicin function [111, 184]. Such *emtA* positive *E. faecium* has also been found to harbor vancomycin, gentamicin, tetracyclines, and erythromycin and streptogramin resistance genes in chickens [88]. *Enterococcus faecium* isolated from broiler chickens treated with virginiamycin demonstrated resistance to quinupristin-dalfopristin, supporting previous observations on the induction of quinupristin-dalfopristin resistance from the use of virginiamycin [185, 186]. Ciprofloxacin, macrolides, penicillin and tetracycline resistant *E. faecium* strains were isolated from broilers fed bambermycin, penicillin, salinomycin, bacitracin, or a salinomycin/bacitracin combination [4]. Moreover, MDR *E. faecium* and *E. faecalis* isolates showing resistance phenotypes and genotypes against bacitracin, erythromycin, tylosin, lincomycin, streptomycin, gentamycin, tetracycline and ciprofloxacin were reported in commercial broiler [97].

As there are a limited number of studies that investigated resistance profiles of important Gram-positive bacteria in organic and ABF poultry production, it is imperative to broaden this topic of research. Miranda et al. [176] reported lower prevalence of resistant *Enterococcus* spp. from organic chickens compared to conventional chickens. Moreover, prevalence of MDR *Enterococcus* spp. was higher in conventional chickens compared to organic chickens [176]. In South Korea, organically-produced poultry demonstrated less prevalence of resistance to ciprofloxacin and erythromycin (commonly used in veterinary medicine) compared to conventionally-produced poultry [187]. Interestingly, Kilonzo-Nthenge et al. [148] reported increased total *Enterococcus* spp. but less AMR *Enterococcus* spp. in organic chicken compared to conventional chickens, predominantly showing resistance to streptomycin and erythromycin.

#### *Staphylococcus spp.*

*Staphylococci* are widespread in nature and comprise of coagulase-positive and coagulase-negative species able to induce minor and major infections in poultry and human [188]. The coagulase-positive *Staphylococcus aureus* can cause infections such as omphalitis, pneumonia and arthritis [189]. The treatment of *S. aureus* infections becomes difficult due to the emergency of multiple antibiotic resistant isolates including MRSA resulting from AMU in both animal and human. In Belgium, MRSA also resistant to antimicrobials including tylosin, amoxicillin, trimethoprim-sulfamethoxazole, lincomycin, tetracycline, and colistin were isolated from broiler [190]. Penicillin, tetracycline and ciprofloxacin-resistant *S. aureus* strains have been reported in different conventional broiler production operations in Korea along with four MRSA isolates from three different operations [191]. South Africa investigations of antibiotic resistance by Amoako et al. [192] in *S. aureus* from poultry and their products using the “Farm to Fork” approach showed a prevalence of 31.25% (*n* = 120/384) of *S. aureus* in analyzed samples: farm (40), transport (15), abattoir (30), and retail point (35) [192]. The authors reported that isolates were resistant to tetracycline (61.7%), penicillin (55.8%), erythromycin (54.2%), clindamycin (43.3%), doxycycline (36.7%), ampicillin (34.17%), moxifloxacin (30.8%), amikacin (30.83%), trimethoprim-sulfamethoxazole (30.0%), and levofloxacin (23.3%) with 100% of isolates being susceptibility to tigecycline, teicoplanin, vancomycin, nitrofurantoin, chloramphenicol, and linezolid [192]. In 2006, *S. aureus* isolated from poultry demonstrated increased resistance against antibiotics compared to *S. aureus* isolates from 1970s [193]. Multidrug-resistant *S. aureus* strains in farm could contaminate chicken meat during processing. Accordingly, MDR *S. aureus* isolated from raw poultry meats were reported, with a highest resistance prevalence being observed towards β-lactams, macrolides, quinolones, and fluoroquinolones [177]. There was significantly more doxycycline-resistant *S. aureus* from conventional poultry meat than from organic poultry meat [27]. From conventional bioaerosols, coagulase-negative *S. xylosus* isolates resistant to nalidixic acid, novobiocin, penicillin, oxacillin, ampicillin, lincomycin, tetracycline, erythromycin, bacitracin, and streptomycin were observed [194]. These resistant isolates harbored *tetK* (tetracycline), *linA* (lincomycin), *ermB* (erythromycin) and *blaZ* (β-lactam) genes.

#### *Clostridium perfringens*

Isolates of *C. perfringens* resistant to bacitracin, penicillin, streptomycin, tetracyclines and gentamicin have been reported in poultry [195]. Moreover, *C. perfringens* isolated from conventionally produced broilers demonstrated resistance to tetracycline and bacitracin, and intermediate resistance to lincomycin [196]. Bambermycin resistance also was observed in *C. perfringens* from poultry, pig, and cattle in Belgium farms [197]. Antimicrobial resistance in *Clostridium* including *C. perfringens* and other anaerobes has been reviewed recently [198]. High prevalence of AMR were reported in *C. perfringens* isolated from broiler chickens in Egypt, namely neomycin, colistin, pefloxacin, trimethoprim-sulfamethoxazole, gentamicin, streptomycin, lincomycin, oxalinic acid, erythromycin and spiramycin [199]. Despite the importance of *C. perfringens* on poultry health, there are a lack of studies that investigated the prevalence of AMR *C. perfringens* in organic and ABF poultry production. Thus, more research is warranted to better understand the impact of organic and ABF poultry production on AMR in *C. perfringens.*

## Conclusion

The poultry industry is rapidly growing due to market and consumer’s demand. However, adopting alternative poultry production practices to improve bird health and performance while decreasing AMU is imperative due to AMR concerns. In poultry production systems, complex environmental and genetic factors could contribute to the prevalence and spread of AMR and their related ARGs despite existence of correlations between AMU and AMR. In ABF and organic poultry productions, several antibiotic alternatives and vaccines are currently being applied. However, cost-effective benefits for most of the alternatives to antibiotics in poultry remain to be established. These alternative products appear to have pleotropic activities including antimicrobial, antioxidant, immune stimulatory and anti-inflammatory actions indicating that more investigations are required to determine their mechanism of action both against bacteria including their AMR profiles and birds. Overall this review indicates that AMR was present in poultry production systems that did not use any antimicrobials but a significant lower prevalence than in conventional poultry. However, more studies to investigate AMR in organic/ABF poultry production need to be done. Furthermore, understanding how feeding programs impact the commensal gut microbiota, pathogenic bacteria, and AMR will help guide dietary and bird health management practices. Accordingly, extensive efforts using integrative One Health approaches are imperative to breakdown the emergence and spread of AMR in poultry.

## Data Availability

Not applicable.

## Abbreviations

AMU: Antimicrobial use
AMR: Antimicrobial resistance
GIT: Gastrointestinal tract
ARG: Antimicrobial resistance genes
NE: Necrotic enteritis
ABF: Antibiotic-free
NAE: No antibiotic ever
RWA: Raised without antibiotics
CIA: Critically important antibiotics
CFC: Chicken farmers of Canada
FOS: Fructooligosaccharides
MOS: Mannanoligosaccharides
SCFA: Short-chain fatty acids
LBBP: Low-bush blueberry pomace
NSP: Non-starch polysaccharides
PBPs: Penicillin-binding proteins
NAG: N-acetylglucosamine
NAM: β-(1–4)-N-acetylmuramic acid
BMD: Bacitracin methylene disalicylate
BACN-Z: Zinc-bacitracin
ExPEC: Extraintestinal pathogenic *E. coli*
APEC: Avian pathogenic *E. coli*
MDR: Multi-drug resistance
MRSA: Methicillin-resistant *Staphylococcus aureus*
VRSA: Vancomycin-resistant *Staphylococcus aureus*
VRE: Vancomycin-resistant *Enterococcus faecium*
